# Fast-track surgery decreases the incidence of postoperative delirium and other complications in elderly patients with colorectal carcinoma

**DOI:** 10.1007/s00423-013-1151-9

**Published:** 2013-12-13

**Authors:** Yitao Jia, Guixing Jin, Shangwei Guo, Bin Gu, Zujian Jin, Xing Gao, Zhongxin Li

**Affiliations:** 1 Oncology Department, Hebei General Hospital, Shijiazhuang, Hebei China; 2 Psychiatric Department, The First Hospital of Hebei Medical University, Shijiazhuang, Hebei China; 3 Department of Surgery, The Forth Hospital of Hengshui City, Hebei, China; 4 Second Department of Surgery, The Forth Hospital of Hebei Medical University, No. 169, Tianshan Road, Shijiazhuang, Hebei China 050000; 5 Gynecology and Obstetrics Department, The Yiwu Affiliated Hospital of Zhejiang University, Yiwu, Zhejiang China

**Keywords:** Delirium, Elderly, Colorectal carcinoma, Fast-track surgery, Interleukin-6

## Abstract

**Objective:**

This study aims to investigate the role of fast-track surgery in preventing the development of postoperative delirium and other complications in elderly patients with colorectal carcinoma.

**Methods:**

A total of 240 elderly patients with colorectal carcinoma (aged ≥70 years) undergoing open colorectal surgery was randomly assigned into two groups, in which the patients were managed perioperatively either with traditional or fast-track approaches. The length of hospital stay (LOS) and time to pass flatus were compared. The incidence of postoperative delirium and other complications were evaluated. Serum interleukin-6 (IL-6) levels were determined before and after surgery.

**Results:**

The LOS was significantly shorter in the fast-track surgery (FTS) group than that in the traditional group. The recovery of bowel movement (as indicated by the time to pass flatus) was faster in the FTS group. The postoperative complications including pulmonary infection, urinary infection and heart failure were significantly less frequent in the FTS group. Notably, the incidence of postoperative delirium was significantly lower in patients with the fast track therapy (4/117, 3.4 %) than with the traditional therapy (15/116, 12.9 %; *p* = 0.008). The serum IL-6 levels on postoperative days 1, 2, and 3 in patients with the fast-track therapy were significantly lower than those with the traditional therapy (*p <* 0.001).

**Conclusions:**

Compared to traditional perioperative management, fast-track surgery decreases the LOS, facilitates the recovery of bowel movement, and reduces occurrence of postoperative delirium and other complications in elderly patients with colorectal carcinoma. The lower incidence of delirium is at least partly attributable to the reduced systemic inflammatory response mediated by IL-6.

## Introduction

Although the conventional surgical treatment is still the most utilized and effective treatment for colorectal malignancy, the procedure may lead to prolonged hospital stay, increased medical costs, and medical resource overuse due to the aggressiveness of the approach and the high risk of developing postoperative nutrition disorders. Patients are often dissatisfied with their functional recovery and the incidence of complications is 20 to 30 % [1, 2]. Although the incidence of conventional complications such as anastomotic leakage has decreased with the improvements of surgical instruments and techniques, postoperative psychiatric complications such as delirium are frequent, particularly in the elderly (≥70 years). As reported previously, about 10–50 % of advanced-age patients undergoing surgical treatment may develop delirium postoperatively [3-5]. Postoperative delirium is a reversible and fluctuating acute brain syndrome characterized by changes in consciousness, orientation, attention, memory, sensory perception, thinking, emotion, and volition. The occurrence of this condition may lead to prolonged hospital stay and unfavorable prognosis, and may develop into chronic brain syndrome (dementia) [6].

Although the mechanism of delirium remains unclear, it has been clearly demonstrated that multiple factors are involved [7, 8]. Besides advanced age, a recent study showed that systemic stress and inflammatory response might play an important role in the development of this condition [9, 10]. It has been reported that the serum levels of an inflammatory biomarkers, including interleukin-6 (IL-6), were positively correlated to the incidence of delirium [11]. Moreover, the highest level of IL-6 was present during postoperative delirium [12]. Therefore, reduction of the perioperative stress and inflammatory response may minimize the occurrence of delirium.

It has been found in several clinical trials that fast-track surgery (FTS) not only facilitates the physical rehabilitation of the patients with colorectal malignancies, but also prevents upregulation of proinflammatory cytokines including IL-6, with reduced stress response and inflammation [13, 14]. Moreover, Krenk et al. have shown that delirium was not observed in fast-track hip and knee arthroplasty in elderly patients [15]. However, there is little data on whether FTS can prevent or protect elderly patients with colorectal carcinoma from developing delirium after colorectal surgery. In the present randomized trial, we studied whether FTS could prevent or reduce the occurrence of postoperative delirium as well as other complications in elderly patients with colorectal carcinoma and evaluated the role of IL-6 in postoperative delirium.

## Patients and methods

### Patients and study design

**Fig. 1:**
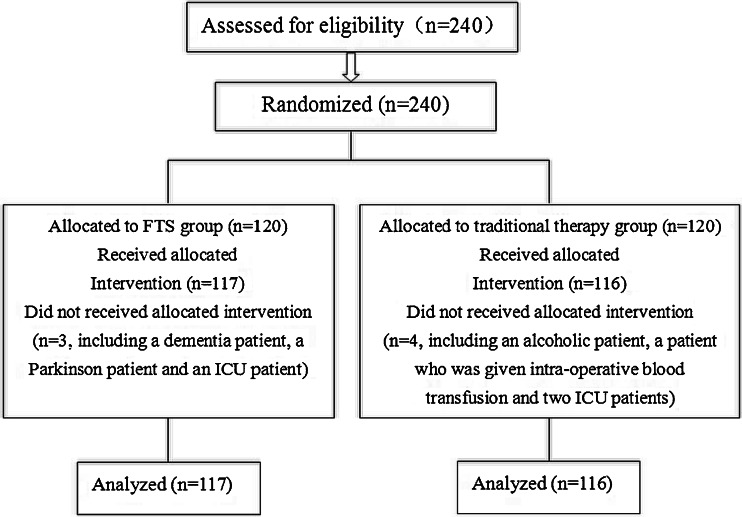
Flow diagram of study

A total of 240 elderly patients with colorectal carcinoma, with ages ranging from 70 to 88 years (mean, 75.18 years; 150 men and 90 women), admitted to the Fourth Hospital of Hebei Medical University for open curative resection between 2008 and 2011, were included in the present study. These patients were randomly assigned into the traditional therapy group (*n* = 120) and the FTS group (*n* = 120). Block randomization of the patients was computer generated. Eligible patients were randomly assigned to each group in a 1:1 ratio (Fig. 1).

Patients with a history of dementia, Parkinson’s disease, alcohol intake of ≥250 g/day, long-term use of sleeping pills or anxiolytics, and those who received anesthesia within the past 30 days were excluded from the study during the initial screening. Those enrolled patients who were subsequently given intraoperative blood transfusion or were admitted to the intensive care unit (ICU) for further treatment after operation were also excluded from analysis (Fig. 1).

Of the 240 study participants, 115 were diagnosed with colon cancer and 125 with rectal cancer. Patients in the two groups had comparable baseline characteristics including gender, age, site of lesion, TNM Classification of Malignant Tumours (TNM staging), and surgical procedure (Table 1). In the current study, a preoperative routine cranial magnetic resonance imaging (MRI) scan was performed for all the patients in both groups, and a repeat MRI scan performed for those who developed postoperative delirium to exclude cerebrovascular stroke or other central nervous system (CNS) conditions as the etiology of delirium.

**Table 1:** Clinical data of the fast track surgery (FTS) and traditional therapy groups (*n* = 233)

Characteristics	FTS (*n* = 117)	Traditional (*n* = 116)	*p* value
Age	75.66 ± 4.18	74.78 ± 4.01	0.054
Gender			
Male	76	70	0.467
Female	41	46	
Site of lesions			
Colon	57	58	0.845
Rectum	60	58	
TNM staging			
I	13	19	0.633
II	52	47	
III	37	38	
IV	15	12	
Surgical operation			
Colectomy	52	53	0.930
Dixon	39	36	
Miles	26	27	
Hypertension	26	33	0.275
Diabetes	11	14	0.511

This study was approved by the Ethics Committee of the Fourth Hospital of Hebei Medical University. All patients signed a written informed consent before participation.

### Patient perioperative monitoring

The length of hospital stay (LOS) and time to pass flatus were documented. The incidence of complications including pulmonary infection, urinary tract infection, intestinal obstruction, anastomotic leakage, heart failure, and deep venous thrombosis (DVT) were also monitored and documented. All patients were followed up and all parameters were obtained from all patients. The diagnosis of postoperative delirium as the focus of this study is described below.

### Perioperative management of the traditional and FTS group

The perioperative managements for patients of both groups including preoperative preparation, anesthesia, pain control, and postoperative managements are compared and summarized in Table 2. The management of the FTS group differed from the traditionally managed group in the several ways: (1) bowel preparation with oral purgatives instead of a mechanical enema; (2) thoracic epidural anesthesia and postoperative analgesic maintenance via the epidural catheter (ropivacaine, 2 mg/ml maintained for 48 h, controlled to 6–10 ml (12–20 mg) per hour and opium-derived agents were excluded); (3) no nasogastric tube insertion; (4) no drainage tube placement with the exception of low rectal anastomosis; (5) water was allowed from 6 h postoperation, liquid diet in the morning and semiliquid diet at noon and evening of the first and second postoperative days (POD) with regular diet on POD 3; (6) early urine catheter withdrawal (at POD 1–2); and (7) early out-of-bed mobilization (i.e., walking).

**Table 2:** Comparison of fast-track and traditional perioperative care protocols

	Traditional	Fast track
Preoperative preparation	Liquid diet for 3 days	Oral purgatives
	Mechanical enema(1time/day) for 3 consecutive days	No mechanical enema
	Fasting at 8 h	Normal meal until 6 h before surgery
	Drink deprivation 4 h before surgery	Normal carbohydrate drink until 2 h before surgery
	Routine nasogastric tube insertion	No nasogastric tube insertion
	Oral antibiotics administration for 3 days	No antibiotics
Anesthesia	General	Thoracic epidural
Pain control	Fentanyl0.25 mg/ml	Ropivacaine2mg/ml
	Midazolam0. 5 mg/ml	
	Nefopam1.0 mg/ml	via PCEA
	via PCIA	
	For 48 h	For 48 h
		Opium-derived agents were excluded
	Routine drainage tube placement	No routine drainage tube placement
Postoperative management	Diet: liquid diet intake after recovery of bowel movement	Diet: water was allowed from 6 h postoperation, liquid diet in the morning and semiliquid diet at noon and evening of the first and second postoperative days, regular diet on POD 3
	Urinary catheter withdrawal at 3 to 5 days	Urinary catheter withdrawal on POD 1–2
	Out-of-bed mobilization at 3 to 5 days	Out-of-bed mobilization on POD 1

### Diagnostic criteria of delirium

The mental status and cognitive function was evaluated in accordance with the Delirium Rating Scale-Revised-98 (DRS-R-98) [16]. The DRS-R-98 evaluates the cognitive domain of delirium by recourse to specific evaluations for attention, orientation, short-term memory, long-term memory, and visuospatial ability. DRS-R-98 has a high sensitivity (91–100 %) and specificity (85–100 %) for detection of delirium [16]. Delirium was defined as the total score ≧18.

The DRS-R-98 scoring was performed on the day of admission and then daily from POD 1 for 5 days. The presence or absence of delirium was evaluated by a psychiatrist and a nurse based on the criteria specified in DRS-R-98.

### Serum IL-6 determination

Fasting peripheral venous blood samples (5 ml) were collected 1 day preoperatively and on POD 1, 2, and 3. The blood samples were centrifuged at 3,000 rpm for 5 min, and the sera obtained were preserved at −20°C for later use. The serum IL-6 levels were determined using sandwich ELISA with the reagents purchased from Invitrogen, Camarillo, CA, USA. The results are expressed as mean ± SD.

### Statistical analysis

Statistical analyses were performed using SPSS 15.0 software package. The measurement data are represented as mean ± SD. The intergroup comparison was performed using the Wilcoxon rank-sum test. A chi-square test was used for the analysis of numeration data. *p* < 0.05 was considered as statistical significant.

## Results

Of the elderly colorectal cancer patients, 240 were enrolled in this randomized trial. The patients were randomly assigned to the FTS group and the traditional management group. The age, gender, site of lesions, TNM staging, surgical procedures, and co-morbidity including hypertension and diabetes between the two groups were comparable, without statistically significant differences (Table 1).

Four patients were excluded from the traditional group: two patients received an intraoperative blood transfusion and the other two were admitted to ICU because of pulmonary infection. Three patients were excluded from the FTS group: one received an intraoperative blood transfusion, and the other two were admitted to ICU because of pulmonary infection. Thus, a total of 233 patients including 116 patients in the traditional group and 117 patients in the FTS group were included this study (Fig. 1).

The mean LOS of the FTS group and traditional therapy group was 9.01 ± 1.75 and 13.21 ± 1.32 days, respectively (*p* < 0.001). The time to pass flatus in the FTS group was significantly shorter than in the traditional therapy group (48.50 ± 9.59 vs. 77.66 ± 7.18 h; *p* < 0.001). On the POD 1, the level of serum albumin in the FTS group was higher than that in the traditional therapy group (28.05 ± 2.82 vs. 26.26 ± 4.12; *p* < 0.001). Meanwhile, the glucose in the FTS group was lower than that in the traditional therapy group (8.30 ± 2.49 vs. 10.25 ± 2.43; *p* < 0.001). No significant difference of liver or renal function was observed between the two groups after operation (Table 3).

**Table 3:** Comparison of postoperative recovery and complications between the FTS and traditional group

	FTS (117)	Traditional (116)	*p* value
LOS (day)	9.01 ± 1.75	13.21 ± 1.32	<0.001
Functional recovery			
Time to pass flatus (h)	48.50 ± 9.59	77.66 ± 7.18	<0.001
Serum albumin (g/L)	28.05 ± 2.82	26.26 ± 4.12	<0.001
Glucose (mmol/L)	8.30 ± 2.49	10.25 ± 2.43	<0.001
ALT (IU/L)	34.65 ± 12.25	34.88 ± 11.82	0.738
AST (IU/L)	30.43 ± 10.78	29.47 ± 10.40	0.356
Cr (μmol/L)	77.05 ± 23.80	75.11 ± 25.04	0.675
BUN (mmol/L)	5.63 ± 3.60	5.62 ± 3.08	0.831
Complications (cases)			
Infection of incision	6	8	0.570
Pulmonary infection	6	19	0.006
Urinary infection	5	13	0.047
Anastomotic leakage	3	2	1.000
Intestinal obstruction	4	6	0.736
Heart failure	4	13	0.022
DVT	4	7	0.340

*ALT* alanine transaminase, AST aspartate transaminase

### Incidence of complications

The postoperative complications were followed and documented based on Clavien–Dindo Classification system, including infection, intestinal obstruction, anastomotic leakage, heart failure, and DVT [17]. The incidence of postoperative delirium is described in the next section.

Six pulmonary infections were observed in the FTS group, significantly fewer than the 19 cases in the traditional therapy group (*p* = 0.006). The incidence of urinary infections was lower in FTS group than that in the traditional therapy group (5 vs. 13; *p* = 0.047). The incidence of heart failure was much higher in the traditional therapy group than that in the FTS group (13 vs. 4, *p* = 0.022). However, no statistical differences were found in the incidences of incision infection, bowel obstruction, anastomotic leakage, and DVT between the two groups. The results are summarized in Table 3.

### Incidence of postoperative delirium

Using the DRS-R-98 scoring system, a total 19 cases of postoperative delirium were observed, 15 in the traditional therapy group (12.9 %) and 4 in the FTS group (3.4 %, *p* **=** 0.008) (Table 4). In the traditional therapy group, nine incidents of delirium were observed at POD 1, five at POD 2, and one at POD 3, while for the FTS group, three incidents of delirium were observed at POD 1 and one at POD 2 (Table 4).

**Table 4:** Incidence of postoperative delirium in the FTS and traditional group

POD	FTS	Traditional	*p* value
POD 1	3	9	0.073
POD 2	1	5	0.211
POD 3	0	1	0.498
Total (% of analyzed cases)	4 (3.4 %)	15 (12.9 %)	0.008

Repeat MRI scanning for those with postoperative delirium did not show any CNS changes (data not shown). This indicates that postoperative delirium was not due to apparent organic CNS involvements, e.g., cerebrovascular stroke, after the surgery [18]. The treatments for delirium included intensive nursing care, and for, severe cases, haloperidol (0.5 mg) was intramuscularly administered and repeated if necessary at an interval of 30 to 60 min. The delirium-related symptoms resolved in all 19 patients after the treatments.

### Serum IL-6 level

**Fig. 2:**
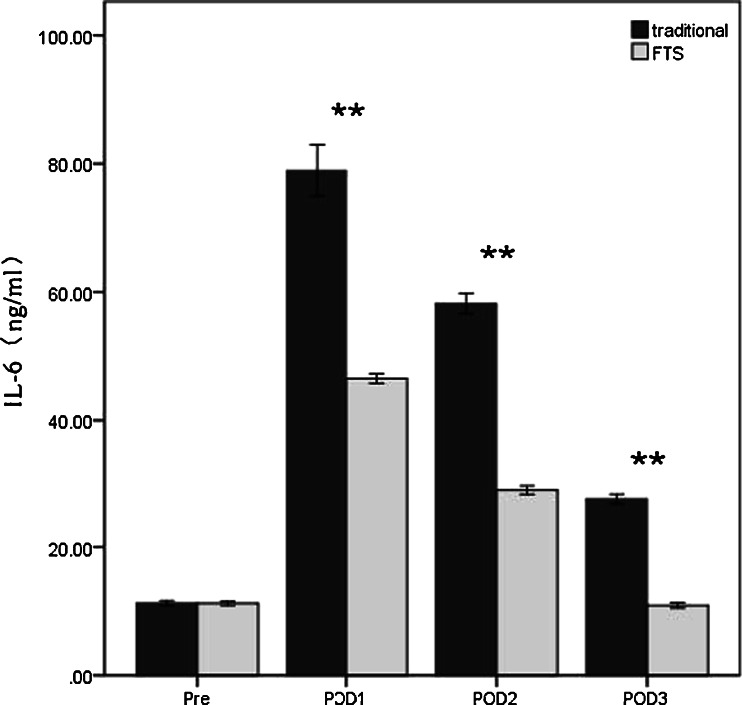
Serum IL-6 levels in the FTS group and traditional group. **p < 0.001 traditional vs. FTS group. *Pre* preoperation, *POD* postoperative day

As proinflammatory cytokines particularly IL-6 have been reported to be involved in the development of postoperative delirium [10, 19-21], we determined the serum IL-6 levels in both the FTS and the traditional groups. The preoperative baseline IL-6 levels in both groups were very similar. For both group, the serum IL-6 level peaked at POD 1, followed by a rapid decline thereafter. This IL-6 peak correlated with the highest incidence of delirium on POD 1 (Fig. 2). Although the IL-6 level in FST group decreased to approximately the baseline level by POD 3, the IL-6 level of the traditional group was still well above the baseline level (Fig. 2). The serum IL-6 levels in the traditional therapy group at POD 1, 2, and 3 were all significantly higher than those in the FTS group (*p* < 0.001 in all 3 POD days). Together, these results showed that enhanced IL-6 level correlated with the development of postoperative delirium in the elderly patients undergoing colorectal surgery.

## Discussion

In the present randomized trial, patients of advanced age (≥70 years) with colorectal carcinoma were treated with either FTS (117 patients) or traditional approaches (116 patients). The patients managed with FTS had significant shorter LOS and fast recovery of bowel movement (Table 3). Importantly, there was a decrease in the incidence of postoperative complications including pulmonary infection, urinary infection, and heart failure (Table 3), which is consistent with previous reports [22, 23]. Notably, FTS treatment was also associated with a decreased frequency of postoperative delirium (Table 4). FTS did not cause the same degree of increase in serum level of the proinflammatory cytokine IL-6 (Fig. 2).

Delirium is an acute brain syndrome most commonly seen in the elderly, particularly in those with underlying organic brain pathology and/or functional impairments [24]. The incidence of postoperative delirium varies depending on the surgical and anesthetic approaches used and ranges from 10 to 24 % in gastrointestinal surgery [4, 5, 25, 26].

It is well established that postoperative delirium is a multifactorial event. Predisposing factors include age; comorbid disease; and cognitive, visual, and hearing disorders [7, 8, 27, 28]. In our trial, all the patients in both groups were of advanced age (≥70 years) and were more prone to developing delirium after surgery. The precipitating factors include the type and extensiveness of the surgical procedure, anesthetic protocol, opioid pain killer usage, inflammatory response, infection, pain, sleep disturbance, nutritional condition, and electrolyte homeostasis [29, 30]. Since the highest frequency of delirium occurred early on POD 1, we believe that preoperative preparation, surgical approach, anesthesia, and pain control protocols were among the most important factors, although other subsequent complications (e.g., postoperative infection) might also play a role most likely later during the course. In our study, patients in both groups underwent similar open colorectal surgery (Table 1), the extensiveness of the surgery and trauma incurred in both groups were similar and unlikely to be a contributing factor. Pain and sleep disturbance reported in both groups were comparable (data not shown), so they were not a likely explanation for the discrepancy of incidence of delirium between the two groups. It has been reported that early feeding in open colon resection in the elderly resulted in shorter LOS and reduced postoperative morbidity [31], so it is possible that early feeding in the FST group might be contributing to the overall lower postoperative complications including delirium. The two groups received different preoperative preparation (Table 2). In the traditional group, mechanical enema and nasogastric tube insertion were applied, while in the FTS group, only oral purgatives was given and no nasogastric tube was used. It is conceivable that these less-invasive procedures used in the FTS group could potentially decrease the stress response, and be a factor in the lower occurrence of postoperative delirium.

A major difference in the management of patients in the two groups was the different anesthesia approach, pain control, and drug selection (Table 2). It is unclear whether general anesthesia per se might play a role in postoperative delirium, but it was possible that it might cause more extensive systematic stress response than epidural anesthesia applied in the FTS group [32, 33]. In addition to anesthesia methods, intraoperative and postoperative medication may affect the mental status of patients [34, 35]. For patients with traditional therapy, opioid drugs including morphine, dolantin, and fentanyl that were used during general anesthesia and postoperative pain management might contribute to the occurrence of delirium [36]. For FTS, opioid drugs were avoided. Epidural anesthesia and analgesia may block the sympathetic response that has been reported to be involved in delirium [37]. The analgesic agent used in FTS group was ropivacaine, which can alleviate moderate to severe postoperative pains effectively and safely [38]. Together, these measures can help reduce the various stimuli to the patients during surgical injury, minimize postoperative inflammatory reaction, and facilitate functional recovery. Therefore, integrated measures should be applied to prevent elderly colorectal patients from developing postoperative delirium.

Excessive release of proinflammatory cytokines such as TNF-α, IL-1, IL-6, and IL-8 during systemic inflammation can affect brain functions [10] and promote the development of delirium [39]. To investigate whether FTS reduced the incidence of delirium by minimizing the inflammatory response, serum IL-6 levels were also determined. We found that enhanced IL-6 level correlated with the development of postoperative delirium and peaked on POD 1 in the elderly patients undergoing colorectal surgery (Fig. 2 and Table 4). FTS significantly reduced the level of IL-6 increase as compared to the traditional therapy group (Fig. 2). This is consistent with the previous reports that elderly patients who develop postoperative delirium may exhibit an elevated serum IL-6 level [39, 40]. It is well known that IL-6 is an endogenous cytokine released by monocytes, T cells, and vascular endothelial cells and is a major inducer of the inflammation [41]. IL-6 can promote the differentiation of lymphocytes and amplify the inflammatory response leading to tissue damage [42]. In this study, there was a correlation between elevated levels of IL-6 and delirium, both of which were present at reduced levels in the FTS treatment group. The underlying mechanisms for reduced IL-6 level in the FTS patients are multifactorial. Less invasive preoperative preparation (no mechanic enema, no nasogastric tube insertion, etc.), use of epidural instead of general anesthesia, and avoidance of opioids for anesthesia and pain control could all contribute to the reduced level of IL-6 in the FTS group. It has been reported that opioids can stimulate IL-6 production in both animal and human studies [43-47], so anesthesia and analgesia without utilizing opioid drugs may be an important factor for reduced IL-6 levels and reduced postoperative delirium in patients managed with FTS. Although we only examined IL-6 level in the peripheral blood, which may not directly represent the intracerebral conditions accurately, however, it has been shown that IL-6 can readily cross the blood–brain barrier [48, 49].

In summary, FTS shortens the LOS, facilitates the recovery of bowel movement, and reduces occurrence of postoperative delirium and other complications in elderly patients with colorectal carcinoma. The incidence of delirium correlates with the serum IL-6 level. The lower incidence of delirium in the FTS group is likely attributable to the reduced systemic stress and inflammatory response mediated by IL-6.
